# Altering the Course of Technologies to Monitor Loosening States of Endoprosthetic Implants

**DOI:** 10.3390/s20010104

**Published:** 2019-12-23

**Authors:** João Henrique Cachão, Marco P. Soares dos Santos, Rodrigo Bernardo, António Ramos, Rainer Bader, Jorge A. F. Ferreira, António Torres Marques, José A. O. Simões

**Affiliations:** 1 Department of Mechanical Engineering, University of Aveiro, 3810-193 Aveiro, Portugal; 2 Center for Mechanical Technology & Automation (TEMA), University of Aveiro, 3810-193 Aveiro, Portugal; 3 Associated Laboratory for Energy, Transports and Aeronautics (LAETA), 4150-179 Porto, Portugal; 4 Department of Orthopedics, University Medicine Rostock, 18057 Rostock, Germany; 5 Mechanical Engineering Department, University of Porto, 4200-465 Porto, Portugal

**Keywords:** endoprosthetic implant, loosening detection, bone–implant interface, bone–implant integration state, implant stability, instrumented implant, bioelectronic device

## Abstract

Musculoskeletal disorders are becoming an ever-growing societal burden and, as a result, millions of bone replacements surgeries are performed per year worldwide. Despite total joint replacements being recognized among the most successful surgeries of the last century, implant failure rates exceeding 10% are still reported. These numbers highlight the necessity of technologies to provide an accurate monitoring of the bone–implant interface state. This study provides a detailed review of the most relevant methodologies and technologies already proposed to monitor the loosening states of endoprosthetic implants, as well as their performance and experimental validation. A total of forty-two papers describing both intracorporeal and extracorporeal technologies for cemented or cementless fixation were thoroughly analyzed. Thirty-eight technologies were identified, which are categorized into five methodologies: vibrometric, acoustic, bioelectric impedance, magnetic induction, and strain. Research efforts were mainly focused on vibrometric and acoustic technologies. Differently, approaches based on bioelectric impedance, magnetic induction and strain have been less explored. Although most technologies are noninvasive and are able to monitor different loosening stages of endoprosthetic implants, they are not able to provide effective monitoring during daily living of patients.

## 1. Introduction

Millions of joint replacements surgeries are performed per year worldwide [1,2,3], as a result of an increasing trend in the burden of musculoskeletal disorders, registered both in developed and emerging countries over the last decades, and now accounting for ~20% of global disability [4]. Although the total joint replacements have been recognized among the most successful surgeries of the last century, the current implant failure rate exceeds 10%, which highlights the inability of today’s implant technology for revision-free performance [5,6]. Indeed, important advances in implant technology have emerged throughout the last 20 years [3,7,8,9,10], but no significant reduction of revision rates have been reported [1,2,3,11]. In fact, these have raised through these years, as reported in most of the orthopedic registers [6,12]. The societal problem is even more relevant when the current and future revision burdens, mainly in young (less than 65 years of age) and active patients, are considered. Approximately 30% of the overall patients are currently young (35% of increase in the last decade), and this percentage is expected to exceed 60% in the next decade [13,14,15]. Besides, surgical revisions are usually more complex and significantly invasive, and the probability to undergo a second revision is five- to sixfold higher after the first revision [16,17]. An effective implant technology must then be developed with ability to fulfill the highly demanding lifetime requirements, ideally to eliminate or reduce the need of revision surgery [1,3,18].

On one hand, a significant increasing use of uncemented fixations has been observed worldwide [19]. Initial fixation of uncemented implants is more difficult to establish when compared to cemented fixation, as it requires both initial mechanical stability and effective biological response to the implant. Uncemented technology is mainly implanted in younger and/or active patients for longer implant survival. [20]. The relevant deterioration risk of cemented fixations in the mid and long-term also supports the decision-making in favor of the bone–implant interface [20]. Nevertheless, the uncemented fixation is more prone to induce bone loss due to stress shielding, a mechanical phenomenon resulting from different mechanical stimuli patterns delivered to the periprosthetic bone stock occurring after implant insertion [18,20]. Revision rates related to stress shielding-induced bone loss can exceed 50%, with incidences confirming implant loosening among the most common causes indicating bone replacement [1,21]. An accurate control of the factors modulating the bone–implant integration process is, then, mandatory to establish an asymptomatic and stable long-term fixation [20,22,23,24,25]. On the other hand, it should be noted that better mobility and reduced post-operative pain seem to be achieved by cementing implants [26]. Although stress shielding also occurs with cemented implants, uncemented fixations are more prone to induced bone loss due to this mechanical phenomenon [20]. Nevertheless, although good stability can be achieved by cemented fixations at short-term following arthroplasty, the mid- and long-term fixation of cemented implants can deteriorate the cement–implant and/or cement–bone interfaces, increasing the risk of aseptic implant loosening [20]. Consequently, improved outcomes of cemented technologies are expected in patients aged 65 years or older [27].

In this paper, we review all relevant studies focused on alternative technologies with those provided by imaging methods to monitor the implant loosening. Included are those incorporated inside instrumented active implants or those operating extracorporeally as adjuvant technologies. To date, conventional monitoring of loosening states is performed by imaging methods, such as radiography, arthrography, scintigraphy, stereophotogrammetry, among others [28]. Although these methods are considered accurate techniques to detect loosening states of both cementeless and cemented implants, the clinical follow-up can only be carried out in clinical laboratories, and thus the monitoring cannot be established throughout the daily life of patients. However, future personalized medicine requires technology innovation to trigger the development of advanced implantable devices to simultaneously perform intensive monitoring and actuation operations, to avoid implant failures [18,29,30]. This new technological trend is emerging to implement multifunctional and intelligent orthopedic implants comprising feedback control systems with the ability to enhance bone growth when loosening states are detected [21,29]. The concept of instrumented implant is an unquestionably disruptive concept that aims to optimize the performance of implants [18,21,29,31]. These are instrumented active technologies incorporating biophysical delivery systems, monitoring systems, wireless communication, and self-powering systems, such that their operation can be controlled by clinicians [18,21,29,30,31]. For example, this concept can be applied in hip prostheses by incorporating stimulation systems inside the implant (to deliver electromagnetic or mechanical stimuli, among others, to bone structures around the implants), near the proximomedial region where bone loss often occurs, as the stress distribution is usually reduced in this region following arthroplasty [18]. Therefore, this work also aims to contribute towards the design of a new era of highly sophisticated implantable medical devices.

## 2. Methods

### 2.1. Literature and Dataset Search

The literature search was carried out by identifying relevant studies in four databases: Scopus, Web of Science, PubMed and IEEE Xplore. Eleven search terms were used: “loosening”, “stability”, “fixation”, “failure”, “detectio”, “monitoring”, “bone”, “joint”, “prosthesis”, “implant”, and “arthroplast”. Advanced boolean searches were conducted according to the following logical formula (adapted according to the syntax of each database); (loosening OR stability OR fixation OR failure) AND (detection OR monitoring) AND (bone OR joint) AND (prosthesis OR implant) AND arthroplasty. The search was extended by reviewing the section “References” of all papers fulfilling the eligibility criteria. The search was completed in April 2019.

### 2.2. Screening of Studies

Studies were screened according to the following inclusion criteria. (i) English language; (ii) types of publication: journal paper, conference paper, and book (chapter); (iii) subject areas: medicine, engineering, physics, and materials; and (iv) publication year: up to 2019. Duplicated references were removed.

### 2.3. Eligibility Criteria

**Figure 1:**
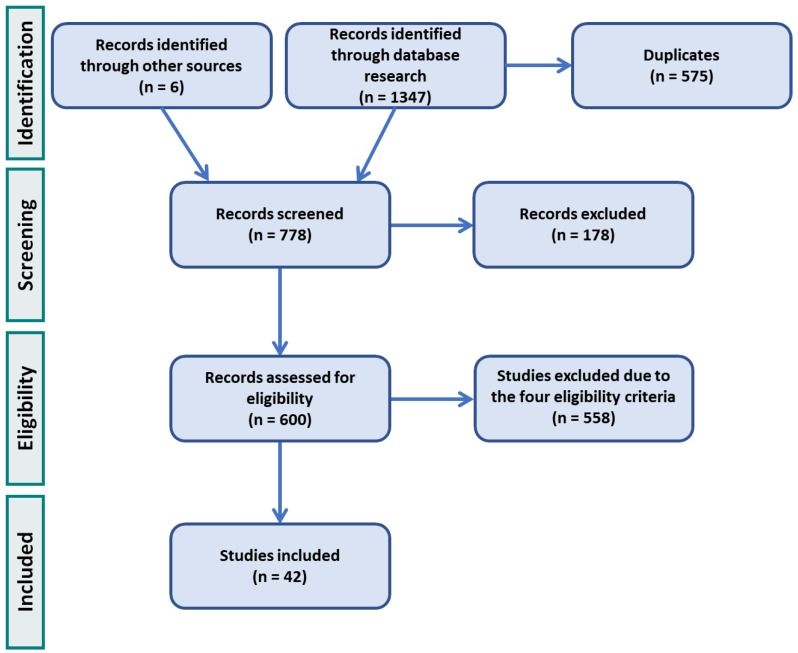
Process of search selection.

Relevant publications were selected according to four criteria: (1) Monitoring technologies not requiring medical imaging methods; (2) Intracorporeal and extracorporeal adjuvant technologies with ability to monitor implant loosening (including those technologies incorporated within instrumented bone implants); (3) Monitoring technologies to monitor the implant–bone interface with in vitro or in vivo validation; (4) Monitoring technologies for both cementless and cemented implants fixations. First, all titles and abstracts were analyzed to assess if the publications fulfilled the eligibility criteria. An additional selection was carried out by assessing the full text of the publication. The number of included publications was reduced to forty-two according to these criteria (Figure 1).

### 2.4. Data Items

Specific features of monitoring technologies were extracted and analyzed from the selected papers: (i) the excitation required for the system operation (system input); (ii) the outcome related to loosening states (system output); (iii) electromechanical components required for each monitoring technology; (iv) location of these components; (v) the ability to monitor the different loosening states, as well as the characteristics of the output signal that allow to distinguish them; (vi) the in vitro and in vivo validation tests already performed by each technology; and (vii) the regularity and flexibility of the technology operation.

### 2.5. Data Analyses

The monitoring methods were categorized in five approaches according to their system outcomes: vibrometry, acoustics, bioelectric impedance, magnetic induction, and strain. An intensive analysis to each of these approaches is presented, first enumerating the existing methods and technologies already developed (the “Input/Output” nomenclature will be referred), and then identifying the most significant limitations of each method. A general analysis of the monitoring technologies is finally addressed. The technology description was carried out by identifying the key components.

## 3. Results

### 3.1. The Vibrometric Approach to Monitor Implant Loosening States

#### 3.1.1. Monitoring Methods and Technologies for Cementless Fixations

**Figure 2:**
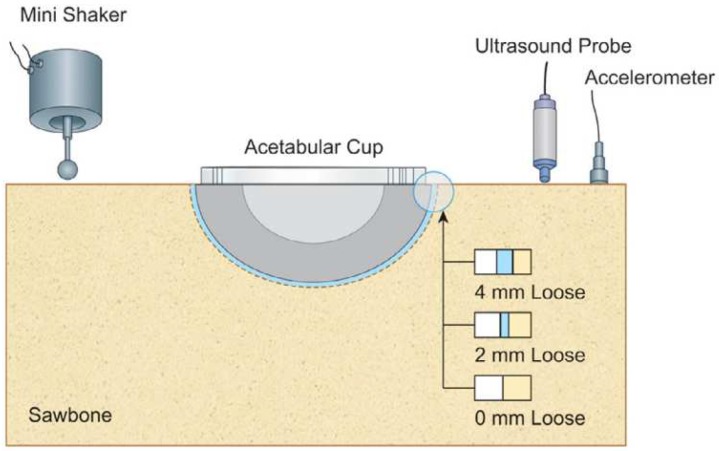
Depiction of the technology T1-L2 of the 1st method of uncemented fixations for both vibrometric and acoustic approaches, illustrating a mini-shaker providing the input excitation signal, the reading components (ultrasound probe and accelerometer), and the acetabular cup. Reprinted from [33] with permission from Elsevier.

**Figure 3:**
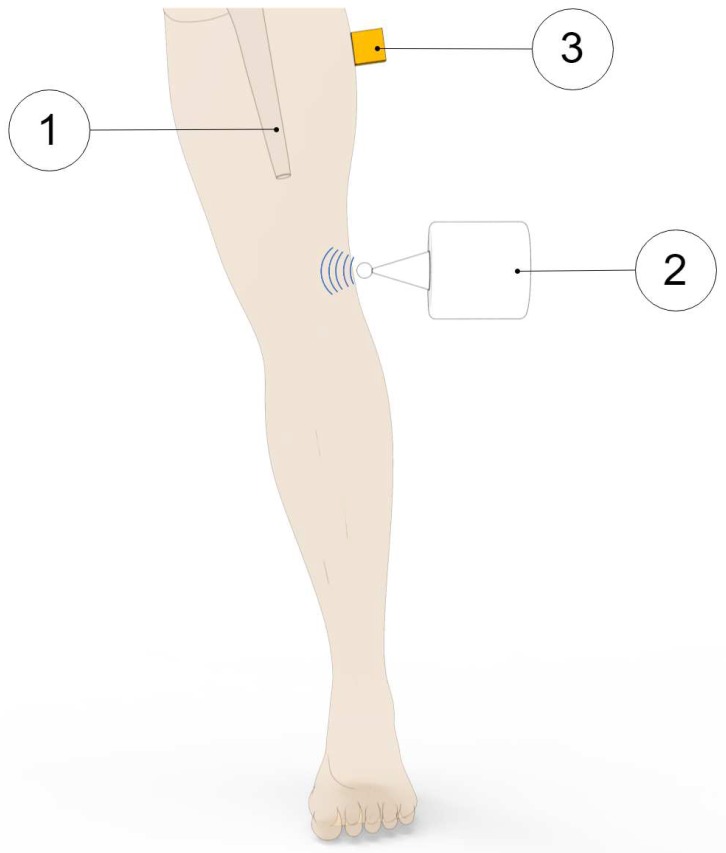
Illustration of the 1st method (extracorporeal mechanical excitation/extracorporeal mechanical signal) for vibrometric cemented and cementless fixations: (1) implant, (2) vibrator providing the input excitation, and (3) extracorporeal accelerometer measuring the resulting vibration from the implant–bone system (or implant–cement–bone). Figure depicting a hip implant case.

**Figure 4:**
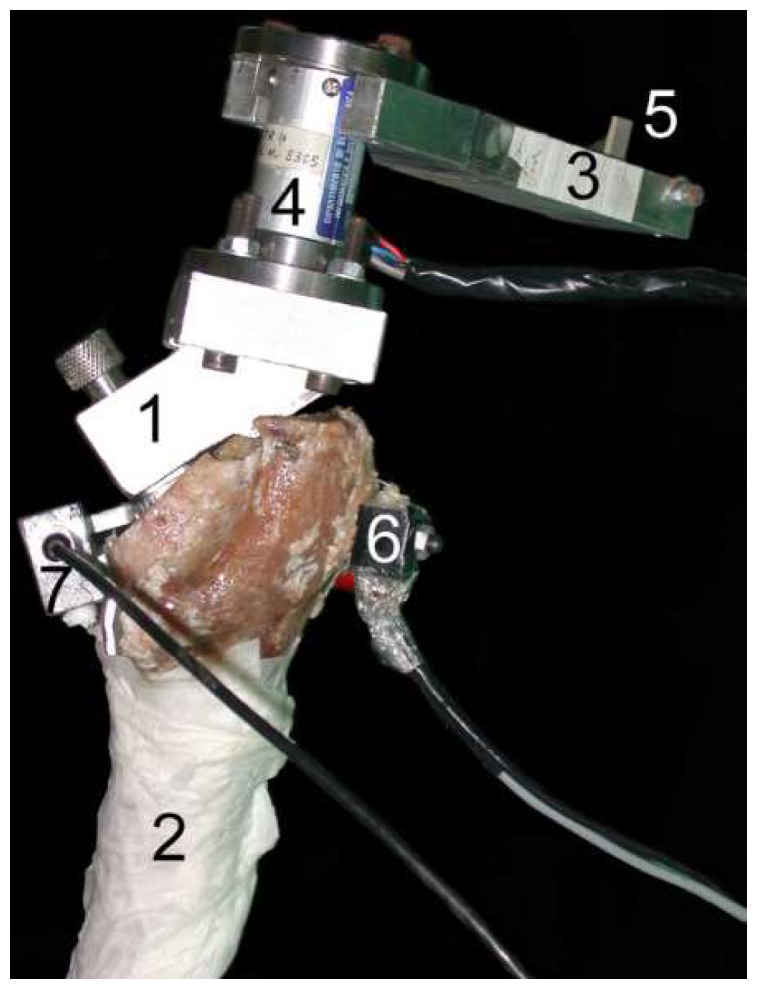
Illustration of the intraoperative technology developed by Varini et al. (T1-L5 of the cementless fixations for vibrometric.) Described by the authors as (1) stem holder, (2) femur, (3) handle to deliver the torque, (4) load cell, (5) component providing the system excitation, and (6) the accelerometer. Reprinted from [38] with permission from Elsevier.

Three methods and ten technologies were already proposed (Table A1, Appendix A):

Method 1: Extracorporeal mechanical excitation/extracorporeal mechanical signal. Eight technologies implemented this method:

(T1-L1) Georgiou and Cunningham [32] designed a noninvasive technology to diagnose loosening of total hip replacements (stem and acetabular component). They use an extracorporeal shaker (excitations up to 1000 Hz) located in the knee (or near the distal femur condyle) and an extracorporeal accelerometer on the hip. The stability assessment of total hip replacements is performed by monitoring the waveform distortion (presence of harmonics) of the output acceleration signals. Only secure loose states can be detected. Loose implants are detected in three scenarios: (i) five or more harmonics, (ii) harmonics with amplitude higher than 50% of the fundamental frequency, and (iii) two or more resonant frequencies. The bone–implant integration failures are noticed in a large frequency range (up to 2000 Hz), and harmonics can emerge exceeding 100 Hz apart from the fundamental frequency.
(T1-L2) Alshuhri et al. [33,34] also proposed a totally noninvasive technology to detect loosening of the acetabular component in total hip replacements. An extracorporeal shaker, in the femoral lateral condyle, and two extracorporeal accelerometers, in the iliac crest and greater trochanter, are required to monitor acetabular cup loosening, which is analyzed by computing the harmonic ratios (relative magnitude of the first harmonic to the fundamental frequency) in the output signal. The mechanical excitation is delivered in the 100 to 1500 Hz range and acetabular loosening is detected if any harmonic ratio is observed. Different fixation scenarios can be identified (authors analyzed two loosening states), as they are correlated to different harmonic ratios. The loosening is distinguished in a large frequency range (up to 1000 Hz) and harmonics can be more than 100 Hz apart from the fundamental frequency. This technology is illustrated in Figure 2.
(T1-L3) Rieger et al. [35] proposed a technology to detect failed implant integration of total hip replacements (femoral stem and acetabular cup) by delivering mechanical excitation on the knee, by an extracorporeal shaker (100–2000 Hz), and subsequent identification of shifts in the resulting resonance frequency of the output vibrations measured by three accelerometers (medial condyle, greater trochanter, and iliac crest) extracorporeally localized (illustrated in Figure 3 with only one accelerometer). Failures are detectable at frequencies below 1000 Hz, but the frequency shifts are in the 2 to 111 Hz range, even though the larger the resonance frequency the larger the frequency shifts. The measures in the ilium only provided frequency shifts (3–22 Hz) for excitations of ~200 Hz. This technology only reports two integrations states (secure or loose), although it allows to differentiate states of stem–cup combinations.
(T1-L4) The research team of Rieger et al. [36] also developed an alternative technology to detect loosening of hip endoprostheses. The mechanical excitation is extracorporeally provided by an array of piezoelectric actuators arranged on a spherical cap to drive shock waves (characterized by an approximation to a Dirac delta function: short rise time, high amplitude, and short pulse width around few μs). The mechanical pulses are delivered from the lateral knee condyle, the greater trochanter, and the iliac crest. These are the same locations where three accelerometers were extracorporeally allocated to allow analyses to shifts in the resonance frequency. This technology allowed to determine significant shifts in the 4 to 847 Hz range (most of them higher than 100 Hz) between 386 Hz and 847 Hz, and can be used to distinguish different states of stem–cup combinations, but only differentiate secure or loose integration levels.
(T1-L5) Lannocca et al. [37] and Varini et al. [38] engineered a medical device customized to measure stability intraoperatively. It is attached to the implant system and comprises an extracorporeal piezoelectric system (piezoelectric cantilever vibrator based on a ceramic multilayer bender) to provide excitations in the 1200 to 2000 Hz range. An extracorporeal accelerometer located on the greater trochanter is also required to analyze the primary stability, performed by monitoring shifts in the resonance frequency. The identified threshold for differentiating between stable and quasi-stable implants is a frequency shift of 5 Hz. Different shifts provide data concerning different primary stabilities. The technology of Varini et al. is represented in Figure 4.
(T1-L6) Lannocca et al. [37] and Varini et al. [38], using the same medical device to deliver the mechanical excitation, proposed to include a displacement transducer (LVDT) to track the primary stability by measuring implant–bone micromotions. Micromotions higher than 150 μm are an intraoperative indication of implant instability. Different micromotions are used to distinguish different primary stabilities.
(T1-L7) Pastrav et al. [39] also contributed towards the perioperative monitoring of fixation of total hip endoprostheses. A shaker and a mechanical impedance head are attached to the prosthetic neck. They found frequency response patterns shifted to the right, and sustained increases as a function of the stiffness increase between successive insertion stages.
(T1-L8) Jiang, Lee, and Yuan [40] tested a noninvasive technology to distinguish between failed (by wear and malalignment) and normal total knee replacements. An isokinetic dynamometer is used to impose extracorporeally excitations based on knee flexion–extension motions (up to 67°/s), as well as an accelerometer positioned on the skin covering the patella. Early and late stages of failure can be identified by analyzing the spectral power ratios of dominant poles of a transfer function representing the vibration signals. The physiological patellofemoral crepitus signals are also able to detect wear of knee components. A threshold, using the average spectral power ratio of dominant poles, was found to identify implant failures. Besides, interface failures are detected by spectral power ratio decreases for frequencies lower than 100 Hz.

**Figure 5:**
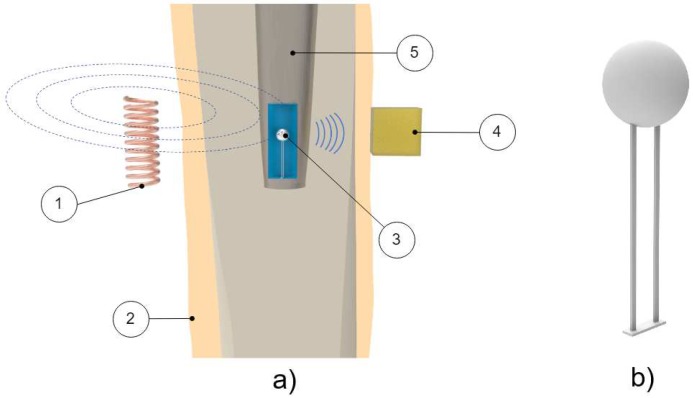
Depiction of the magnetic oscillator method (T2-L1 of cementless fixation of vibrometric technologies). (**a**) Schematic of components: (1) Extracorporeal coil providing movement to the oscillator; (2) Human tissue; (3) Oscillator housed inside the implant; (4) Extracorporeal accelerometer used to measure the resulting vibration from the oscillator’s impact; (5) Implant. (**b**) Detailed illustration of the oscillators.

Method 2: Extracorporeal magnetic induction/extracorporeal mechanical signal. Only a single technology established this method:

(T2-L1) Ruther et al. [41,42] provided an innovative technology based on intracorporeal mechanical excitation driven by extracorporeal magnetic induction and extracorporeal acceleration sensing (depicted in Figure 5). They developed an oscillator–implant system in which one or more magnetic spherical oscillators, attached to a flat spring, are embedded into the implant near the stem walls for detection of loosening features in several endoprosthetic devices (including total hip and knee replacements). The vibrational excitation is inductively provided by a coil extracorporeally, producing a magnetic field that imposes collisions of the oscillators with the implant walls, which causes the propagation of vibrations along the adjacent tissues surrounding the implant that can be measured by an accelerometer externally located at the skin surface. The measurement of the resulting accelerations signals and subsequent computation of the frequency shift in the output signal, as well as the central frequency in the resultant spectrum, allows prediction of the differing loosening locations and stages (press fit, slight loosening, and significant loosening). Shifted frequencies around 300 Hz and 400 Hz allow the detection of slight loosening and significant loosening, respectively, although better results were achieved using the central frequency as an indicator, as these frequencies always varied more than 1000 Hz for any loosed scenario under analyses. These authors also demonstrated an effective change in the central frequencies (exceeding 500 Hz) for different measurement locations, apart from a geometric reference in the range extended from the 5 to 124 mm range (distance in the three-dimensional space). Besides, they found longer transient periods for unstable fixations.

Method 3: Intracorporeal mechanical excitation/extracorporeal mechanical signal. Only a single technology established this method:

(T3-L1) Glaser et al. [43] developed a noninvasive technology to monitor the performance of hip joint implants within the bone–implant interface. An intracorporeal excitation is delivered by the implant displacement during dynamic movements of patients. Mechanical vibration can be detected by placing two accelerometers: one at the greater trochanter and the other on the anterior superior iliac spine. The acetabulum–femur separation is identified by a high-frequency sound, which is originated by the impact caused when the femoral head slid back into the acetabular component.

The proposed methods were specifically designed for medical analyses related to hip and knee joint implants, although they hold potential for implementation in other bone implants. A deeper analysis of these methods and technologies reveals significant findings, as follows. The most explored method was the one requiring extracorporeal mechanical excitation and extracorporeal mechanical signal (eight technologies out of ten). Most methods (nine out of ten) established the use of extracorporeal excitation systems to monitor bone–implant integration; among them, most technologies (eight out of ten) require a mechanical excitation to drive the monitoring system. The mechanical excitation was neither undervalued by Ruther et al. [41] nor by Glaser et al. [43]; instead, such an excitation was used as an intermediate process between the primary excitation and measured outcome. Harmonic excitations are delivered by six technologies, but shock waves and magnetic induction were also used. Note that eight of the technologies perform monitoring operations using extracorporeal accelerometers. Concerning the interface monitoring of total hip implants, note that technologies were developed for the detection of both femoral stem and acetabular cup loosening. Note that three technologies (out of nine) are able to identify different loosening stages, and six of them are limited to a monitoring operation on the no-loosening-loosening basis; however, no technology was designed to analyze bone–implant integration states along distinct bone–implant locations, even though theoretical analyses were already conducted towards the design of instrumented implants with such ability [44]. Concerning technologies for total knee systems, their operation was focused on the detection of interface failures. Another relevant matter concerns the effective monitoring period: most monitoring systems (seven out of ten) were designed for postoperative sensing, and only three technologies were customized for intraoperative monitoring operations. Data processing operations were mainly conducted by analyzing the resulting shifts in the resonance frequency, but analyses of the waveform distortions, harmonic ratios, central frequencies, bone–implant micromotions, spectral power ratios, and transient periods were also considered. The sensitive band was usually found in the 1500 to 2500 Hz range [45]. The vibrometric approach was validated both in vitro and in vivo, although some technologies (six out of ten) have not yet been validated in vivo. Furthermore, their performance was not measured in terms of measure accuracy.

#### 3.1.2. Monitoring Methods and Technologies for Cemented Fixations

Two methods and eight technologies were already proposed (Table A2, Appendix A), as follows.

**Figure 6:**
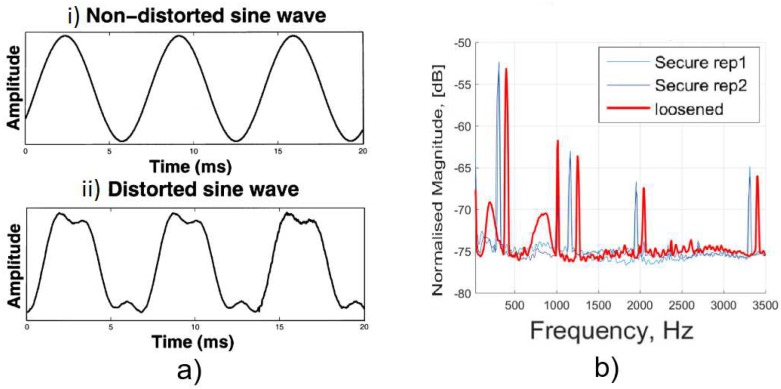
(**a**) Graphic showing the distinction of waveform in the cases of loose and fixed implants. The fixed case output signal (i) resembles the input frequency driving the system. In contrast, for the loose case (ii), signal distortion can be observed. Adapted from the work in [51] with permission from Elsevier. (**b**) Graphic showing the effect of a loose implant in a frequency analysis. The loose implant (in red) can be characterized (by comparison with the fixed, in blue) by an increase in the resonance frequency and by the appearance of harmonics in the output signal. Adapted from the work in [50] under the Creative Commons Attribution 4.0 International License: http://creativecommons.org/licenses/by/4.0/.

Method 1: Extracorporeal mechanical excitation/extracorporeal mechanical signal. Five technologies implemented this method:

(T1-C1) Li, Jones, and Gregg [46] developed a similar technology to T1-L1 (Georgiou and Cunningham [32]), but for cement–bone–implant interfaces. They used a shaker to deliver extracorporeal mechanical vibrations (100 to 1200 Hz) at the distal femur and monitored the output vibration by two extracorporeal accelerometers at the distal and proximal femur. The output signal was analyzed in the same frequency bandwidth as the extracorporeal excitation. Implant loosening was detected by distortion analyses of the output acceleration waveforms, as well as using the number of resonance frequencies (two or more). The authors tested three fixation states: secure, early loosening, and late loosening. Loose implants are characterized by highlighting more than two resonance frequencies and present a distorted output signal in several excitation frequencies. Contrarily, early implant loosening fixation states are not clearly distinguished from the secure state.
(T1-C2) Similarly to Li, Jones, and Gregg [46], Rosenstein et al. [47] developed a method to assess the stability of cemented hip implants. They applied a mechanical excitation provided by an extracorporeal shaker (100 to 1000 Hz) at the lateral condyle while measuring the resulting output vibration, in the same bandwidth, with an extracorporeal accelerometer on the greater trochanter. The tests were only performed with fixed and loosened cemented implants (two loosening states). The loosening was correlated with harmonics in the output acceleration signals, although no specific frequency values were reported.
(T1-C3) The research team of Rowlands, Duck, and Cunningham [48] also developed a method to monitor hip implant loosening. Using an extracorporeal shaker to provide input mechanical vibrations (100 to 1500 Hz) in the distal femur, they monitored response vibrations on the greater trochanter using an extracorporeal accelerometer. Although four fixations states were analyzed (a loosened state, as well as three fixed states), only the results regarding the loose implant were reported. Loosening is observed by analyzing the output signal resonance frequency. The most sensitive band for the driving frequency was found between 100 and 450 Hz. Apart from the loose implant results, no additional data was provided.
(T1-C4) Leuridan et al. [49] developed a technology to assess the fixation state of tibial knee implants. Distinct tests were conducted by varying the measurement region (tibia surface and tibial plate) and using extracorporeal accelerometers to measure the output signals in the 50 to 4500 Hz frequency band. Mechanical excitation was provided by an impact hammer at the tibial plate surface. Four different cement–bone–implant interface scenarios were reported: secure, peripheral loosening, medial loosening, and lateral loosening. The authors used two criteria to process the resonance frequency results: the Modal Assurance Criterion and the Frequency Assurance Criterion; fixation states could be distinguished by the different values given by each criteria. The most sensitive band was found to be above 1500 Hz.
(T1-C5) Arami et al. [50] also provided a technology to detect loosening states of tibial knee implants. An extracorporeal shaker located below the patella (100 mm) was used to deliver mechanical excitations in the 30 to 3000 Hz frequency range. Three extracorporeal accelerometers were used: one was fixed in the vibrator tip, such that the output frequencies can be analyzed in the same range as the input, and the remaining two accelerometers were used to assess the vibration propagation to the tibial implant and were placed in the tibial plate. They assessed two interface states: well-fixed (cemented) and completely loose. Implant loosening is characterized with a new peak emerging in the 750 to 900 Hz range, when compared to a baseline result taken from the well-fixed case. Furthermore, peak shifts of 53.1 ± 13.7 Hz (in the 700 to 1200 Hz range) and 66.2 ± 9.0 Hz (in the 1200 to 2200 Hz range) can be observed. A graphical example of these two loosening indicators can be seen in Figure 6b).

Method 2: Extracorporeal mechanical excitation/intracorporeal mechanical signal. This method was established by three technologies:

(T2-C1) Puers et al. [51] also designed an instrumented hip prosthesis but, differently, housing an acceleration sensor and some additional electronics in the implant head. The implant loosening detection is observed by analyzing the waveform distortion of the output acceleration signal (Figure 6a) when extracorporeal vibrations (100 to 200 Hz) are driven by a shaker placed on the distal end of the femoral bone. Loosened implants are detected by observational verification of non-similarity between the excitation signal and the measured acceleration outcome. Two interface states distinction were reported: secure or loose.
(T2-C2) Marschner et al. [52] incorporated a two-axis accelerometer and supporting electronics inside an instrumented hip implant (distal end of stem) to measure shifts in the resonance frequency of the output vibrations when an extracorporeal shaker delivers a mechanical excitation (500 to 2500 Hz) on the distal femur condyle. This technology to detect loosening of total hip replacements also includes the ability to perform wireless monitoring and to be inductively powered. Two loosening states (proximally loose and proximally secure) can be distinguished in a band within the 1500 to 2500 Hz range. The shift threshold can exceed 300 Hz.
(T2-C3) Sauer et al. [53] developed a similar technology to Marschner et al. [52] by incorporating a three-axis acceleration sensor in the implant head (and some additional electronics) and delivering extracorporeally mechanical excitations (500 to 2500 Hz), and, using a shaker placed at the central part of the femur, the implant loosening detection is observed by identifying shifts in the resonance frequency of accelerations measured inside the implant. Three loosening states were detected in the 500 to 1500 Hz range: maximum, medium, and minimum loosening. These states could be distinguished according to frequency shifts up to 100 Hz (approximately in the 20 to 100 Hz range).

**Figure 7:**
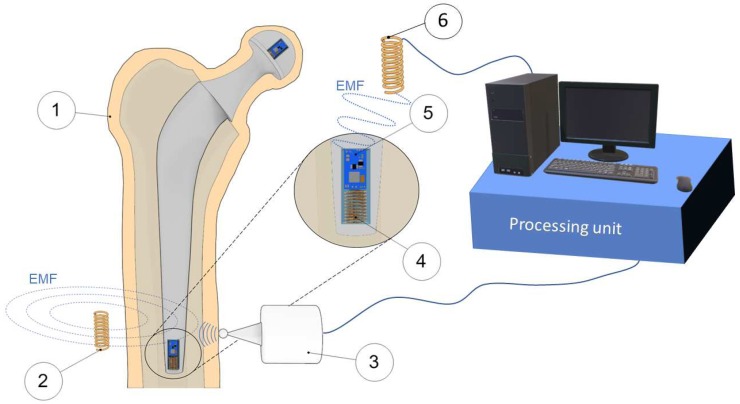
Illustration of the 2nd method (extracorporeal mechanical excitation/intracorporeal mechanical signal) for vibrometric cemented fixations: (1) Human tissue; (2) Extracorporeal coil required to power the system through electromagnetic induction; (3) Extracorporeal shaker providing the input mechanical excitation; (4) Intracorporeal coil used to power the system; (5) Intracorporeal monitoring system. (6) An extracorporeal coil was used to acquire data from the sensor through magnetic induction; afterwards, the data is sent to a processing unit (EMF: electromagnetic field).

As observed, for uncemented fixations, the proposed methods were specifically developed for hip and knee joint implants. Most technologies (five out of eight) were developed employing extracorporeal excitation and extracorporeal sensing (Method 1). The less explored method concerns three technologies (out of eight) with a higher level of sophistication by incorporating sensors within the bone implant (Figure 7 schematizes these technologies). A common feature found in all technologies is the requirement for extracorporeal mechanical excitation. Nevertheless, the intracorporeal components of instrumented implants require inductive powering via extracorporeal coils. Regarding the interface monitoring, six technologies (out of eight) only identified two loosening states (secure or loose), whereas the other two technologies identified several loosening stages (up to three). Similarly to the uncemented technologies, none of these technologies were designed to assess loosening in different locations, and their operation is limited to laboratory testing, hardly adaptable for continuous operation throughout the daily life of patients. The loosening detection is mainly computed by analyzing shifts in the output resonance frequency. However, sensing was also performed by evaluating the harmonic ratio and number of harmonics defining the output signal and also by observing the non-similarity between the input excitation and output measured acceleration. All the established methods were validated in vitro, and only one of them was validated both in vivo and in vitro. No measure accuracy was provided to further analyze the technology performance.

#### 3.1.3. Limitations of Vibrometric Monitoring Technologies

A general limitation of vibrometric methods is the patient-dependent output vibration due to the strong influence of soft tissues surrounding implants on mechanical wave propagation [48]. It is pertinent to emphasize that most of the proposed methods are specifically designed for hip and knee joint implants. The ability of the vibrational approach to monitor the fixation state in hip implants was computationally analyzed (using the finite element method) by Qi, Mouchon, and Tan [45] for cemented fixations and by Pérez and Seral-García [54] for uncemented fixations. Regarding the first model, only reliable loosening detection of cemented hip fixations can be obtained for failure sizes exceeding one-third of the stem length, although some inconclusive data can be provided for failure sizes greater than one-fifth of the stem length. Although effective identification of different states of uncemented fixations can be provided, Pérez and Seral-García [54] only managed to detect differences in the resonance frequency for input frequencies greater than 2400 Hz. In addition, Leuridan et al. [49] also developed a computational model to predict failures on cemented knee implants, namely, to detect loosening of the tibial component. Failure states can be detected when more than 15% of the implant surface is loose on the lateral and medial side.

Concerning the first monitoring method (input: extracorporeal mechanical excitation; output: extracorporeal mechanical signal) for both uncemented and cemented fixations, as extracorporeal technology is required, two mutually exclusive scenarios may occur: (i) the nonstop tracking of fixation states requires the attachment of technologies to the patient’s body, which is uncomfortable and troubles the activities of the patients, or (ii) the circumstantial monitoring implies the inability to obtain effective monitoring data throughout the daily living of patients. Indeed, this issue could be overcome if the monitoring technologies could be designed to be incorporated inside instrumented implants. Nevertheless, the overall components were not designed to be housed within the implants, but only the acceleration sensors and processing systems (as carried out by technologies developed for cemented fixations using the 2nd method). The ability of this noninvasive method to detect where loosening failures are occurring was not demonstrated. Still, the loosening location cannot be accurately detected due to the diffusivity nature of the mechanical excitation, which makes the delivery of different excitations to much closed target regions quite hard to achieve [18]. Most technologies are only able to detect if the implant is loose or the opposite. Five technologies successfully provide more than two levels of detection. Regarding the medical devices engineered to analyze the intraoperative implant stability [38,39], an overall analysis to the bone–implant interlocking (primary stability) along the implant surface cannot be obtained. Besides, as low-frequency shifts are required to identify the stable–unstable threshold, precision and expansive electronic systems are required. Note that only four (out of seventeen) technologies were validated in vivo. Interestingly, an additional technology was reported by Rieger et al. [35] using laser vibrometry, but it is not suitable for clinical practice.

Concerning the second monitoring method (input: extracorporeal magnetic induction; output: extracorporeal mechanical signal) for uncemented fixations, mechanical systems (magnetic oscillators and springs) must be embedded within the instrumented implant to provide an intracorporeal excitation, even if an extracorporeal excitation system is required, similarly to the technologies designed by Puers et al. [51], Marschner et al. [52], and Sauer et al. [53]. This limitation troubles an effective biointegration monitoring throughout the daily living of patients. The use of magnetic induction to provide the primary excitation also demands complex extracorporeal systems to deliver different excitations to different intracorporeal oscillators, and thus a personalized monitoring of target regions along the implant surface will be hard to achieve [18]. The experimental results using the technology developed by Ruther et al. [41] highlight the difficulty to distinguish between different loosening states by analyzing the resonance frequency shifts. Nevertheless, a significant technological breakthrough was also performed by Ruther et al. [44], as they designed an instrumented hip implant to measure several loosening locations, although no experimental results (neither in vitro nor in vivo) were provided. Finally, in vivo tests need to be conducted to demonstrate the clinical potential of technologies from the second monitoring method.

Concerning the second monitoring method for cemented fixations, some components of the technologies developed by Puers et al. [51], Marschner et al. [52], and Sauer et al. [53] must operate extracorporeally, namely, to generate and drive the excitation signals. The miniaturization of the excitation source could be carried out to house the overall technology inside the implant; nevertheless, this technology upgrade would not be enough to accurately identify the regions where varying bone–implant integrations occur. The alternative would be to design extracorporeal mechanical vibration systems much more complex, such that they would be able to deliver excitations to much closed target regions. Although the instrumented implants were designed to incorporate acceleration sensors, microcontrollers, and additional electronics to support sensing and telemetric link, the data processing is extracorporeally conducted. Intracorporeal processing capability will require precise electronics and powerful processing units, as low-frequency shifts must be automatically detected, which will certainly impose a more complex and expensive manufacturing of the instrumented implants. Detection reliability requires the use of additional analyses to measured outcomes (such as the computation of the central frequency, as proposed by Ruther et al. [41]), which, in turn, demands additional processing capability. No results were reported related to loosening states in different regions along the implant surface. Besides, neither technologies comprising electromechanical components incorporated inside the implants were validated in vivo, nor measure accuracies were provided.

The single technology proposed in the third method presents some limitations as well. The need for extracorporeal components makes harder for continuous monitoring. One possibility for overcoming that limitation is attaching the components to the patient, although that makes patient movement cumbersome. Another possibility is to integrate the components (accelerometers) in the implant itself, which will in turn require for a miniaturization of the technology. Despite these scenarios, some difficulties in the measurements may arise. Unwanted noises from different movements or even arising from muscular activity can make the analysis troublesome. Furthermore, different implant materials highly influence the output signal, as shown by the authors. This highlights the necessity of studying each individual combination of types of implants and their materials with the objective of recording the different results, aiming to have a comparable data set of the different frequency patterns.

### 3.2. The Acoustic Approach to Monitor Implant Loosening States

#### 3.2.1. Monitoring Methods and Technologies for Cementless Fixations

Three methods and five technologies were already proposed (Table A3, Appendix B), as follows.

**Figure 8:**
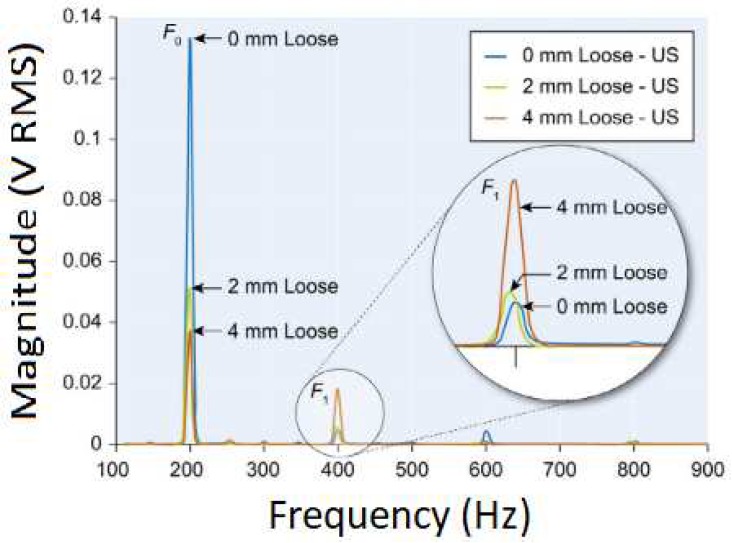
Illustration of the distinction between three implant fixation states: fixed, 2 mm loose, and 4 mm loose. An increase in the harmonics magnitude can be observed with increasing acetabular cup loosening. Adapted from the work in [33] with permission from Elsevier.

**Figure 9:**
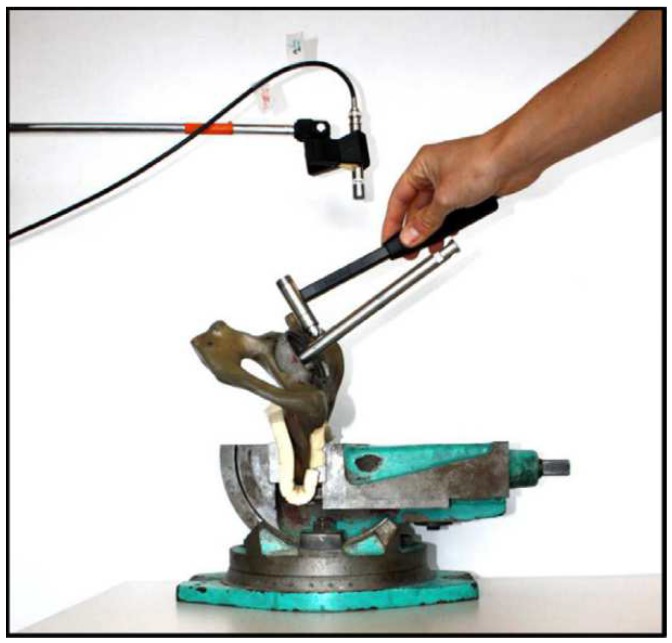
Example of in vitro testing of the T1-L3 technology for uncemented fixations of the acoustic approach. The following components are included; artificial pelvis, microphone (suspended above the artificial pelvis), the metal rod (connected to the acetabular cup), and the hammer (used to drive the system). Reprinted from [56] with permission from Elsevier.

Method 1: Extracorporeal mechanical excitation/extracorporeal acoustic signal. Three technologies implemented this method:

(T1-L1) Unger et al. [55] developed a noninvasive technology to assess the hip implant stability. The extracorporeal excitation was provided by hand hitting the implant with a metallic device. The produced sound was monitored with an extracorporeal microphone attached to the lateral condyle. The implant loosening is distinguished by the response frequency: the resonance frequency increases as stability increases (authors observed increases from 400 to 800 Hz). Different fixation scenarios can also be identified by damping analyses: increasing dampened outputs were observed for increasing stabilities.
(T1-L2) The research team of Alshuhri et al. [33,34] reported an alternative technology to the one previously described in the vibrometric approach to detect the acetabular component loosening for uncemented fixation, also depicted in Figure 2. The only difference concerns the use of an extracorporeal ultrasound probe instead of an accelerometer. Loosening is identified by analyzing harmonic ratios in the output signal. Different loosening scenarios (authors analyzed two loosening states) can be correlated to different harmonic ratios. The ultrasound measurements were performed in the 200 to 1500 Hz range, although the most sensitive excitation frequency was observed in the 200 to 950 Hz range. Note that the output signal presented higher harmonic ratios when compared to the monitoring data obtained using the accelerometer. The ultrasound results are shown in Figure 8.
(T1-L3) Goossens et al. [56] engineered a custom-made technology to monitor the fixation states of the acetabular component of hip implants. The driving excitation input was provided by an extracorporeal hammer by hitting a metal rod connected to the simulated acetabular component. The acoustic outcomes were measured using an extracorporeal microphone, suspended above the experimental setup (approximately 20 cm) (Figure 9). The authors tested several fixation levels which could be distinguished by analyzing shifts in the output resonance frequency. The tests showed differences in the output frequency according to the different components (artificial and cadaveric pelvis) and the shifts were observed ranging from 9 to 248 Hz, which are bone model-dependent.

Method 2: Intracorporeal mechanical excitation/extracorporeal acoustic signal. A single technology implemented this method:

(T2-L1) Glaser et al. [43,57] developed an alternative technology to analyze the output of the previous T3-L1 technology for uncemented fixations in the vibrometric approach. The difference concerns the analysis of the resulting sound emissions from the intracorporeal implant displacement, instead of the mechanical vibrations. Acoustic emissions were recorded with an extracorporeal sound transducer attached on the skin surface in the closest distance to the hip joint interface. High correlation was observed between the data obtained by the sound transducer and the accelerometers.

**Figure 10:**
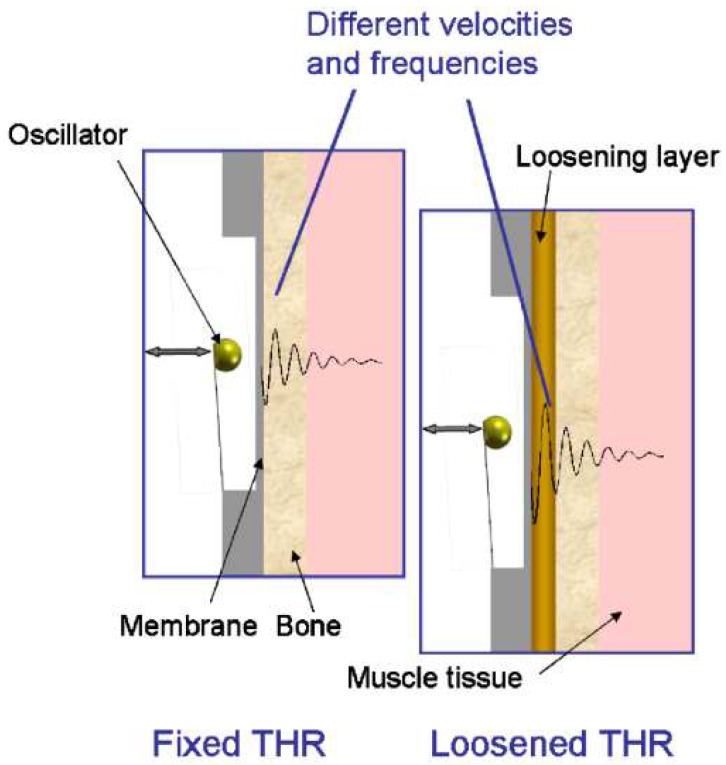
Schematic of the principle behind Ewald et al. and Ruther et al.’s oscillator loosening detection system. It clearly demonstrates the effect of a loose implant in the output signal frequency and intensity [59]. Figure registered under ©2011 IEEE.

Method 3: Extracorporeal magnetic induction/extracorporeal acoustic signal. A single technology implemented this method:

(T3-L1) Ewald et al. [58,59] developed an innovative technology using a similar method to the one proposed by Ruther et al. (technology T2-L1 for uncemented fixations using the vibrometric approach). Similarly to Ruther et al. [58,59], they also incorporated spherical oscillators inside the implant, near the stem wall, attached to a flat spring, which are driven by magnetic induction excitations provided by extracorporeal coils (Figure 10). Unlike Ruther et al. [58,59], they used an extracorporeal microphone to record the resulting sound emission originated by collisions of the oscillators with the implant walls. The output sound emission was recorded on a wide frequency range, namely between 0 and 20 kHz. Different fixation scenarios can be detected (the authors identified up to four) by observing shifts in the output resonance frequency. The resonance frequency of the fixed and loose scenarios is up to 10 kHz apart.

Relative to the system’s input, four out of the five technologies require an extracorporeal stimuli, namely, mechanical vibration or magnetic induction; in contrast, a single technology was developed to record the acoustic emissions originated from the implant’s own motion. Note that all the described studies use extracorporeal sensors to monitor the implant–bone interface state. One may also highlight that two methods minimize patient’s discomfort and contact by using a technology requiring magnetic induction as excitation, or by using a technology that does not require extracorporeal excitation. Similarly to the vibrometric approach, the loosening detection was mainly computed by analyzing shifts in the output frequency (three out of five), although harmonic ratios and the analysis of the amplitude and frequency of the output signal were also assessed. None of the presented technologies are able to identify the regions where loosening occur; however, all the technologies managed to distinguish more than one loosening stage. Regarding the experimental validation, four out of the five technologies only validated their methods in vitro while the remaining one only performed in vivo validation. A common feature shared by all technologies is their inability to provide sensing data throughout the daily living of patients (monitoring limited to laboratory facilities).

#### 3.2.2. Monitoring Methods and Technologies for Cemented Fixations

Four methods and ten technologies were already proposed (Table A4, Appendix B), as follows.

**Figure 11:**
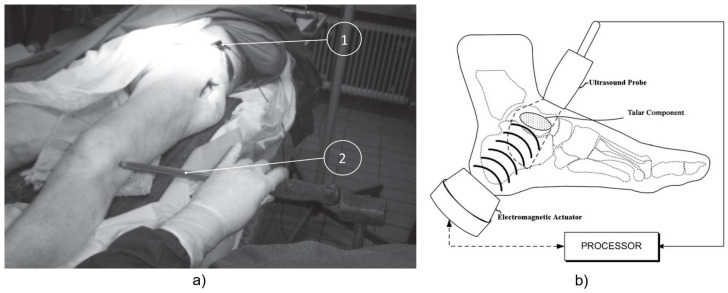
(**a**) Cadaver test performed in the development of T1-C2 technology for cemented fixations of the acoustic approach. In which one can observe (1) the microphone placed on the hip to measure the resulting vibration provided by (2) the impact hammer. Adapted from the work in [55] under the Creative Commons Attribution 4.0 International License: http://creativecommons.org/licenses/by/4.0/. (**b**) Schematic of T1-C3 technology for cemented fixations of the acoustic approach. Reprinted from [60] with permission from Elsevier.

Method 1: Extracorporeal mechanical excitation/extracorporeal acoustic signal. Three technologies implemented this method:

(T1-C1) Rowlands, Duck, and Cunningham [48] engineered a technology similar to T1-C3 technology using the vibrometric approach. The only difference concerns the use of an extracorporeal ultrasound transducer to monitor acoustic signals in the proximal femur. Similar results to the ones provided using extracorporeal accelerometers were observed, although higher magnitude signals can be obtained. Same as before, they only provided results for the loose implant.
(T1-C2) The technology developed by Unger et al. [55] for uncemented fixations (T1-L2) can also be applied in cemented fixations. The driving excitation is provided by hitting the femoral condyle with hammer strikes, and the extracorporeal microphone is attached to the hip. The implant stability is assessed by analyzing shifts in the resonance frequency. At least three distinct loosening scenarios are distinguished: secure, fissured (in the cement), and loose. The detection algorithm includes the analyses to harmonics and damping of the sound outcome, as resonance frequencies and damping increases for increasing stabilities. Different fixation scenarios can also be identified by observing resonance frequencies below 1000 Hz. An in vivo experiment showing the technology operation is illustrated in Figure 11a.
(T1-C3) Dahl et al. [60] developed a technology to quantify different levels of osteointegration of the talar component of total ankle prosthesis (Figure 11b). An extracorporeal actuator (ankle foot orthosis), located around the ankle, drives a mechanical excitation to impose motion to the talar component. The resulted vibration is detected by an ultrasound probe in the skin’s surface. Loose and fixed states are analyzed by computing the ratio of magnitudes of harmonics with the driving frequency: this ratio decreases as the fixations state is improved.

**Figure 12:**
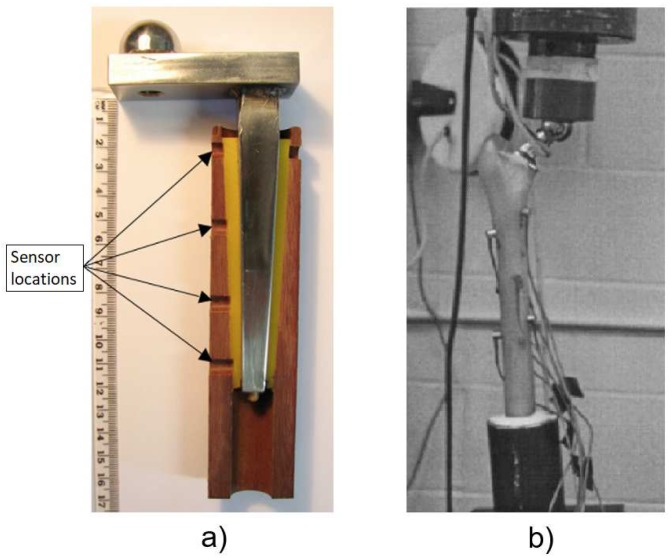
(**a**) Example of in vitro testing of the T2-C5 technology for cemented fixations of the acoustic approach. The used implant, cement, and Tufnol tubing can be observed. Adapted from the work in [65] with permission from Elsevier. (**b**) Experimental set-up used by Qi et al. in the development of T2-C3 technology for cemented fixations of the acoustic approach; the figure shows the implant inserted in the artificial femur, the acoustic emission sensors placed at the femur’s surface, and the loading machine [63]. Figure registered under ©2004 Wiley Periodicals, Inc.

Method 2: Intracorporeal mechanical excitation/extracorporeal acoustic signal. Five technologies implemented this method:

(T2-C1) Davies, Tse, and Harris et al. [61] developed a monitoring technology to assess the cement–stem interface condition after hip arthroplasty. The analysis was focused on monitoring acoustic emissions generated by the cement–metal interface debonding or by cement cracks when the femur is physiologically loaded. An extracorporeal acoustic emission transducer attached to the femur’s mid-surface is required to monitor the acoustic emissions. Different interface stages can be observed by analyzing varying acoustic intensities and waveforms. However, this technology is not able to distinguish acoustic emissions between debonding of interfaces (without cement cracks) and cracks in the cement mantle.
(T2-C2) Roques et al. [62] developed a technology to monitor the fatigue-related cement failures in the bone–cement interface. Two extracorporeal acoustic sensors, up to 70 mm away from each other on the top surface of the cemented device, were used to detect differing acoustic patterns due to crack propagation after static and dynamic loading. Progressive failure is distinguished by analyzing the energy and duration of the acoustic signal output: both increase with the fatigue crack growth. Interestingly, this technology is able to detect the crack location by analyzing the arrival time of the acoustic waves.
(T2-C3) Qi et al. [63] developed a technology to assess cement failures in hip implants using eight extracorporeal acoustic emission sensors, attached along the medial–proximal femoral surface, and dynamically loading the femur. Crack locations can also be detected by measuring the arrival time of the acoustic waves. This monitoring system is able to distinguish the progress of crack formation based on the arrival times, number of events, signal energy, amplitude, and their location distribution. An in vitro test showing the experimental setup is depicted in Figure 12b).
(T2-C4) Gueiral and Nogueira [64] designed a similar monitoring system to Qi et al. [63] (T2-C3) to monitor cement deterioration, but only used three acoustic transducers arranged in a cylindrical disposition at the femur surface. Acoustic events were characterized by their energy, amplitude, and arrival time. Their location can be predicted as well. The authors used the following parameters to characterize the acoustic emissions; amplitude, duration, and number of threshold crossings, although no concrete values were given.
(T2-C5) Mavrogordato et al. [65] also proposed a technology to monitor cement deterioration in hip implants but including the ability to operate with surrounding soft tissues. The excitation is provided by delivering dynamic loading to the hip stem. Four acoustic sensors are externally mounted on the cement mantle along the stem length. The chosen criteria to find relevant acoustic events was based on the energy and rise time of the output signal. This technology is also able to predict the crack location by measuring the arrival time across different sensors. Components and materials used by the authors in the in vitro test can be seen in Figure 12a.

Method 3: Intracorporeal mechanical excitation/intracorporeal acoustic signal. Only a single technology was designed using this method:

(T3-C1) Mavrogordato et al. [65] also engineered a technology using intracorporeal acoustic sensors to monitor the cement–implant interface of hip implants. This method is similar to the previous T2-C5, but the sensors are embedded within the implant stem. The results regarding the intracorporeal sensors showed higher sensitivity in detecting acoustic events when compared to the extracorporeal sensors, as well as minor influence from ambient noise.

**Figure 13:**
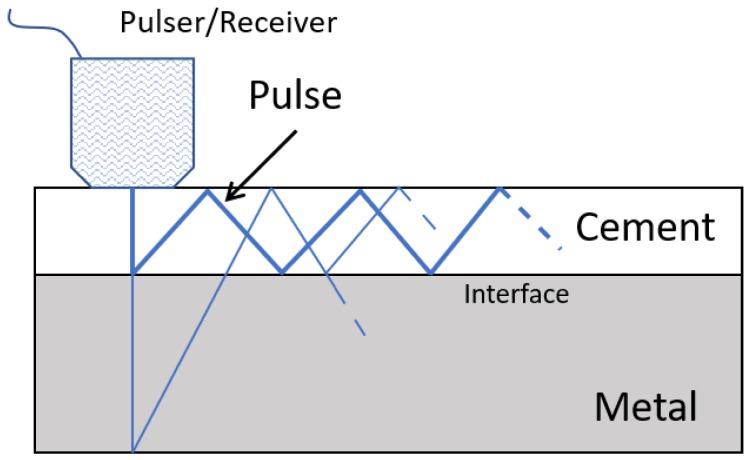
Schematic of the T4-C1 technology for cemented fixations of the acoustic approach. The effect of a bonded/debonded surface on the reflection of the wave and its time of arrival are clearly demonstrated. Based on the work in [66].

Method 4: Extracorporeal acoustic emission/extracorporeal acoustic signal. Only a single technology was tested using this method:

(T4-C1) Davies, Tse, and Harris [66] developed an active acoustic emission technology to assess the cement–implant interface state. The same extracorporeal device—an ultrasonic pulser/receiver—is attached to the femur’s surface. The interface bonding state was identified by emitting an ultrasonic wave through the cement and implant and consequent analyzes to the reflected acoustic signal outcome, namely, the amplitude and arrival time. A bonded interface is characterized by detecting a secondary signal corresponding to the reflection of the wave in the metal surface. In contrast, with a debonded surface, only the primary signal can be observed. Only two interface states were distinguished: bonded and debonded. A scheme illustrating the principle behind the emitter/receiver and the wave reflection is displayed in Figure 13.

Most of the described technologies were developed for hip implants (nine out of ten); only one research group focused their research on the fixation monitoring of an ankle implant. The most explored method requires extracorporeal acoustic emission sensors to monitor the cement mantle integrity (five out of ten): note that most of the proposed technologies, with the exception of one, used extracorporeal sensors to measure the output signal. A wide variety of analysis was conducted to conclude about the interface state. The second and third methods were established by computing the loosening detection by analyzing the acoustic magnitudes, waveform, duration, energy, and rise time. The first method presents similarities, with many of the previously described requiring both extracorporeal excitation and extracorporeal sensors; therefore, stability assessment can be carried out by analyzing harmonic ratios and shifts in the output resonance frequency. In a distinct way, the fourth method analyzed the reflected wave’s magnitude and time of arrival to monitor the cement mantle bonding state. Regarding the interface state monitoring ability, only five (out of ten) technologies can predict the location of acoustic events. Seven (out of the ten) technologies can distinguish between more than two interface states; the other three technologies only managed to distinguish between loose or secure interfaces. All the technologies were validated in vitro, but no in vivo validations were carried out.

#### 3.2.3. Limitations of Acoustic Monitoring Technologies

Similarly to the vibrometric approach, for both cemented and cementless implants, acoustic technologies present a patient-dependent general limitation, due to the influence of the surrounding soft tissues on wave propagation. Another similarity is related to their application: most of the proposed technologies were engineered for hip implants, although some of them were also targeted for knee and ankle implants. Progressive monitoring of the cement integrity was computationally modeled by Qi et al. [63]. Their model was used to assess the 3D locations of the cracks, as well as their dynamic emergence. Although progressive crack monitoring was achieved, the group noted a lack of accuracy in computing the data regarding the crack’s location due to the high sampling rate. A technology resembling the second method (acoustic methodology) for both cemented and cementless fixations was also developed [67,68]. Their technology was implemented for hip implants, such that it comprises four acoustic emission sensors to detect acoustic events originated from the implant’s motion. By progressively monitoring the implant and computing the acoustic emissions, it is possible to detect sounds which can be indicative of the implant’s wear and damage. Although they showed the sensors working principle, a clear correlation between the interface state and the output signal was not reported.

It is pertinent to emphasize that all the proposed acoustic technologies require extracorporeal reading units. This is a significant limitation that shrinks the applicability range and the possibility for continuous interface monitoring, as the electromechanical components attached to the patient’s body cause discomfort and troubles in their daily life, which, excluding the latter possibility, limits the technology’s operation to the laboratory environment, and in turn disregards the interface state dynamics and does not allow timely delivery of therapeutic stimulation [30]. Mavrogordato et al. [65] were the only authors that aimed towards a more sophisticated monitoring system by housing the sensors within the implant’s stem, although their technology also requires external units. The driving mechanism also plays an important role in patient’s comfort and method versatility. Using extracorporeal excitation units, constrains even further the possibility of turning the technology portable. Miniaturization of components could be a feasible solution; nevertheless, the need to carry more than one component attached to the patient’s body imposes an even greater burden. Concerning this matter, the second method (applied to both cemented and cementless technologies) presents an advantage because it does not demand input excitation components, as the driving signals are generated intracorporeally, either from the cement mantle or from the implant’s motion. Furthermore, the use of shakers or hammers may cause pain and discomfort for the patient, although this effect is frequency-related. In addition, by using non-contact excitation systems, such as magnetic induction input sources, the mechanical contact with the patient’s body is minimized, reducing the risk of infections.

Regarding the second method for cementless and cemented fixations, as well as the third method for cemented fixations, a general limitation is shared by the developed technologies: the susceptibility to undesirable or not expected noises. Either from the surrounding tissues or from ambient background sources, there is a number of environmental factors that may compromise the effectiveness of this method. Ambient noises can be avoided by providing a controlled space, although this scenario excludes the possibility of continuous monitoring, which is a requirement for technologies assessing the progressive damage in cement. Other acoustic signals arising from unwanted internal sources can be avoided by using databases of known noises. A research group [67] resorted to using patients with natural hips to provide a baseline of ambient noise characteristics and unwanted vibrations due to the implant motion.

Concerning the fourth cemented method, the estimation of the wave’s reflection will most likely be hard to achieve due to topological nonlinearities of the interface. The authors also stated that the smallest detectable area was half the diameter of the transducer. Furthermore, as this technology only comprises extracorporeal components, it will have the associated limitations as previously explained.

### 3.3. The Bioelectric Impedance Approach to Monitor Implant Loosening States

#### 3.3.1. Monitoring Methods and Technologies for Cementless Fixations

One method and one technology were already proposed (Table A5, Appendix C), as follows.

**Figure 14:**
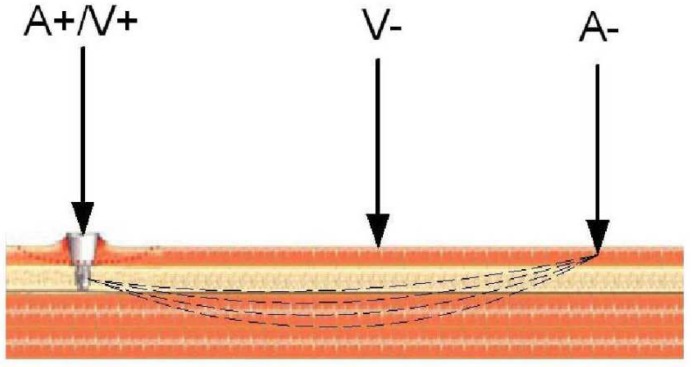
Illustration of the working principle of the single proposed technology in the bioelectric impedance approach. One can see the electrodes placed on the skin surface to generate an alternate current (A+/A−), resulting in a voltage drop (V−) that can be directly correlated to the impedance [70]. Figure registered under ©2007 IEEE.

Method 1: Extracorporeal electrical current/extracorporeal electric potential difference. A single technology established this method:

(T1-L1) Arpaia and Clemente et al. [69,70] used electrical impedance spectroscopy to assess bone–implant integration states. This technology requires two extracorporeal electrodes, which are used to deliver a variable current at the skin’s surface and measure the voltage drop between them (Figure 14). The resulting impedance is correlated to the interface state: the impedance increases for decreasing levels of integration. Furthermore, no information concerning the location of less stable regions is provided.

This technology exclusively utilizes extracorporeal components and thus its use is limited to a laboratory environment. As described, the interface state can be assessed by computing the resulting electrical impedance, and different interface states can be correlated to different impedance values. The identification of areas with lesser bone–implant integration levels was not achieved. Concerning validation, in vitro and in vivo tests were made, although the latter was performed with percutaneous implants.

#### 3.3.2. Limitations of Bioelectric Impedance Monitoring Technologies

Analyzing the single proposed technology, the use of extracorporeal components limits its monitoring ability, because its use is limited to a specific environment (laboratory). Another important limitation is the influence of patient physiology. As this technology relies directly on the tissues response to electrical stimuli, the output signal will vary accordingly to the tissues composition. A possibility to overcome this limitation is to create a database with the typical impedance values according to the patient’s physical characteristics. Even so, due to the complexity of the human’s tissue, this technology may prove difficult to be applied.

### 3.4. The Magnetic Induction Approach to Monitor Implant Loosening States

#### 3.4.1. Monitoring Methods and Technologies for Cementless Fixations

One method and two technologies were already proposed (Table A6, Appendix D), as follows.

**Figure 15:**
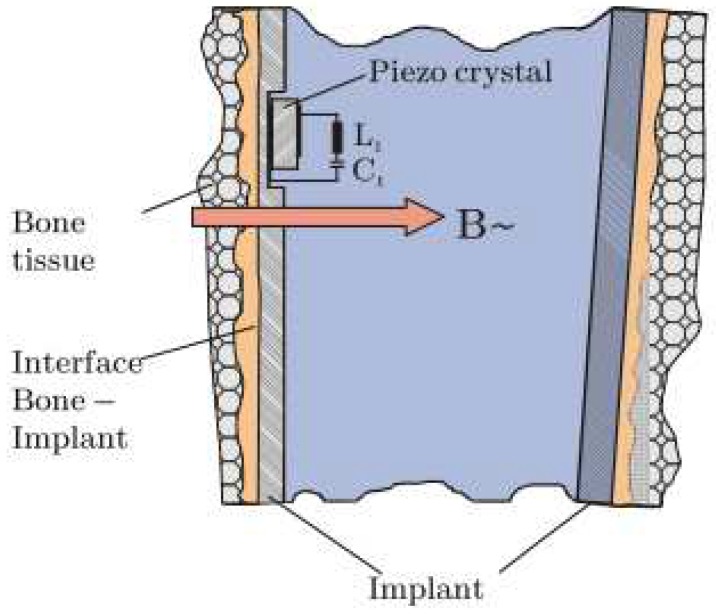
Illustration of the working principle and architecture of the piezo-acoustic method. The crystal is integrated inside the implant’s walls and driven by a magnetic field. The crystal’s vibration dampening is affected by the surrounding tissues [58]. Figure registered under ©2011 IEEE.

Method 1: Extracorporeal magnetic induction/extracorporeal magnetic induction. Two technologies implemented this method:

(T1-L1) Ewald et al. [58] developed a piezo-acoustic method to monitor implant loosening states. The technology comprises a piezo crystal (Figure 15) incorporated within the implant, which vibrates when it is driven by a magnetic field provided by an extracorporeal coil. According to the state of the bone–implant interface, the crystal’s vibration presents different dampening characteristics, which can be measured inductively, throughout an extracorporeal coil. Different interface states can be distinguished by analyzing the output signal amplitude for excitations with a constant frequency (the authors reported a frequency of 83 kHz).
(T1-L2) With a similar technology to T2-L1 of the cementless vibrometric method, Ruther et al. [71] used a different approach to measure the output signal. The same oscillators are driven through magnetic induction provided by an extracorporeal coil, and, instead of reading a resulting mechanical vibration, an extracorporeal coil is used to measure the resultant oscillator velocity caused by the impact. By placing the oscillators in a magnetic field, their displacement induces a current in the extracorporeal coil, which is proportional to their velocity. Different loosening states can be distinguished by computing the oscillator’s velocity after impact.

Both the proposed technologies drive the systems through magnetic induction; moreover, both assessed the output signal also via magnetic induction. The components used for both the excitation and monitoring were extracorporeally positioned. Regarding the detection algorithm, the amplitude of the output signal and velocity of the magnetic oscillators are used. Similarly, both technologies were limited to laboratory analysis and were only validated in vitro.

#### 3.4.2. Limitations of Magnetic Induction Monitoring Technologies

The use of magnetic induction to measure the bone–implant integration requires the use of non-magnetic materials in the interface. This issue can indeed be inconvenient, as the design of multimaterial implants must be considered. Regarding the components, the use of extracorporeal coils limits the application of these methods to a laboratory environment, excluding the possibility for continuous monitoring. Furthermore, the risk of electromagnetic interference emphasizes the need for a controlled testing apparatus. In both technologies, the monitoring ability is limited to the proximity between components. To perform monitoring operation in various regions, several components must be incorporated within the implants along, and near to, the implant’s walls. This feature requires miniaturized components to avoid compromising the implant’s physical integrity.

In a similar way as the vibrometric and acoustic methodologies, this method also relies on the patient-dependent dampening strongly influenced by the tissues condition.

### 3.5. The Strain Approach to Monitor Implant Loosening States

#### 3.5.1. Monitoring Methods and Technologies for Cementless Fixations

Two methods and two technologies were already proposed (Table A7, Appendix E), as follows.

**Figure 16:**
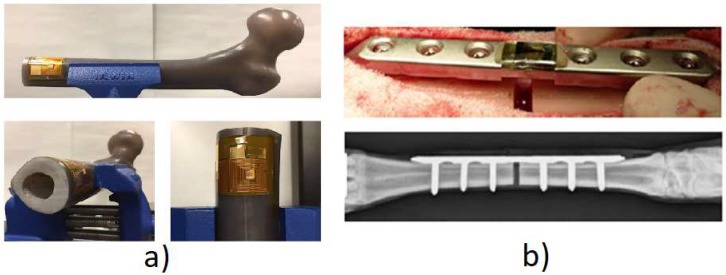
(**a**) Illustration of the flexible capacitive circuit for strain monitoring. The sensors can visibly adapt to the bone tissue structure [72]. Reprinted by Permission of SAGE Publications, Ltd. Copyright ©2019, ©SAGE Publications. (**b**) Fixation plate integrated with the capacitive and resonating sensor (in the middle) [73]. Figure registered under ©2015 Orthopaedic Research Society and published by Wiley Periodicals, Inc.

Method 1: Intracorporeal mechanical loads/intracorporeal bone deformation. A single technology implemented this method:

(T1-L1) Burton, Sun, and Lynch [72] developed a strain sensor to measure bone growth. The technology comprises two cosurface circuits: one for measuring the axial strain, and the other for the radial strain (Figure 16a). Each circuit was connected to a parallel-plate capacitor whose dielectric changes according to strain variations; the second is further connected to a titanium fuse which yields according to a set threshold of radial deformation. Powering and signal reading were achieved through extracorporeal magnetic induction. The changing capacitance values are assessed by monitoring shifts in the output resonance frequency: increasing strains shift the resonance frequency to lower values. This technology is meant to operate in contact with the bone tissue, wrapped around the bone structure.

Method 2: Intracorporeal mechanical loads/intracorporeal fixation plate deformation. A single technology implemented this method:

(T2-L1) McGilvray et al. [73] developed a biocompatible, microelectromechanical technology to track the fracture healing in implantable fixation plates (Figure 16b). It comprises intracorporeal planar capacitors and a resonance circuit incorporated in the implant to monitor variations in physical loading. Changes in the capacitance cause shifts in the resonance response frequency: a decrease in loading increases the resonance frequency. The technology is powered inductively through an extracorporeal antenna which also performs as the receiver of the sensor’s signal.

Both of the proposed technologies in the strain methodology are both driven by mechanical loading, provided by the body’s own weight. Similarly, both technologies output strain is measured through differences in the system’s capacitance, by analyzing the resonance frequency. Regarding the monitoring potential, the technologies managed to distinguish a secure state and several degrees of loosening. Both technologies were validated in vitro, although only one was validated in vivo.

#### 3.5.2. Limitations of Strain Monitoring Technologies

Both of the proposed technologies require extracorporeal powering, which limits the method’s applicability. One solution is to incorporate an intracorporeal power source or attached powering components to the patient’s body, although the latter can be a burden. Furthermore, the reading operation is performed by analyzing the technology response frequency, through magnetic induction, with extracorporeal components. This emphasizes the need for a controlled environment, as electromagnetic noise can interfere with the results. Regarding the first method’s technology, as the circuit is meant to operate in contact with the bone, it can also act as an obstacle for bone integration. Although the technology developed by McGilvray et al. [73] (T2-L1) was designed for fixation plates, the same technology can be applied in other implant technologies requiring bone implant integration.

## 4. Discussion and Conclusions

To our knowledge, no detailed reviews focused on major breakthroughs have been carried out in the scope of monitoring technologies for bone–implant integration sensing so far. Ledet et al. [74] conducted an overview on sensor technology for instrumented orthopedic implants focused on physical parameters that do not provide an effective indication of the bone–implant integration state (characteristics, strength, stiffness, etc). Soares dos Santos et al. [21,75] focused their analyses on all bioelectronic systems (actuation, monitoring, communication, and self-powering) incorporated into instrumented bone implants experimentally validated in vivo, which also confined the measurement technologies to force, moment, and temperature sensors. Karipott et al. [76] also presented an enumeration of some technologies for biomechanical loading and biochemistry monitoring, but only three studies on implant failure detection were briefly reported. Varga et al. [77] published a non-detailed review on medical imaging methods and four alternative monitoring methods, based only on the vibrometric approach, for loosening detection. O’Connor and Kiourti [78] only briefly enumerated three technologies for loosening detection of hip implants. Finally, Ruther et al. [28] summarized the current imaging methods used for diagnostic of post-operative total hip replacement and presented ten methods, using mechanical vibrations and acoustics, for bone–implant loosening detection. However, these studies did not perform detailed analyses to all monitoring approaches already proposed.

This review provides, for the first time, an intensive and critical review of the methodologies and technologies that were developed to monitor the loosening states of endoprosthetic implants. Five different methodologies were proposed, and a total of thirty-eight technologies were developed. Despite these efforts, suitable monitoring systems have not yet been developed, as all developed technologies present significant limitations. Indeed, effective technologies must fulfill the following criteria.

1. Operate noninvasively regarding peri-implant tissues.
2. Allow integration inside implants.
3. Allow stretchable and flexible integration inside implants.
4. Allow their design with different topological structures and for different geometries of the bone–implant interface.
5. Enable controllable and personalized monitoring of target regions on the tissues.
6. Allow follow-up of the bone–implant interface state throughout the daily life of patients.

A comparative analysis of the ability of the monitoring methods (and related technologies) to fulfill these key points is introduced in Table 1. The vibrometric and acoustic methodologies present the widest variety of methods and technologies; in contrast, the bioelectric impedance approach only presents a single technology. Most technologies are able to operate noninvasively (only one technology, related to the strain approach, is not able of such ability). Besides, none of the technologies managed to monitor specific target regions.

Regarding the cementless technologies using the vibrometric approach, the second method fulfills the greatest number of requisites, being distinguished by its ability to be integrated inside the implant with flexible integration. On the other hand, this method has been developed to be integrated into hip implants and, then, it is unknown if it holds potential to be used in other implants. The other missing point is relative to the targeted monitoring of tissues. Although one technology was developed for such goal, its effectiveness was not explored [44]. The cemented technologies of the second method were developed to be incorporated inside the implants and to have the potential to adapt to different topological structures. Concerning the not fulfilled requirements, targeted monitoring and flexible integration are hard to be achieved using the vibrometric approach.

The third method related to acoustic methodology for cementless fixations is the one fulfilling more requisites. Its limitations are similar to those described for the second method of the vibrometric methodology. The other methods were neither developed for integration inside of implants nor to monitor target tissue regions. As in the cemented methods, these methods all present similar limitations, although the third method is distinguished by its ability to be integrated inside implants and the fourth method by its inability to monitor different states of the bone–cement–implant interface. They all lack the flexibility and the potential for monitoring target regions.

The technology based on bioelectric impedance allows to monitor different interface states and ensures that different topological structures and geometries can be designed. Regarding the other features, the incorporation of the technology, as well as targeted monitoring, inside implants can be difficult.

Concerning the only proposed method for the magnetic induction approach, one may notice its inability to adapt to different topologies and geometries, and limitations related to targeted monitoring of tissues. Nevertheless, this method allow the incorporation of the technologies inside the implants, showing some degree of flexibility and the possibility to monitor different states of the implant–bone interface.

Methods developed for the strain approach were both developed as implantable systems, allowing for the conception of different topological designs and the ability to monitor different states of the bone–implant interface, although none of them managed to achieve monitoring of target tissue regions. Regarding the first method, the circuitry and design allow for a very flexible and stretchable integration. Nevertheless, the developed technology is invasive, as it must be attached in contact with bone tissues. The second method can be noninvasively applied ensuring flexibility and allowing a stretchable integration.

This work highlights that significant research efforts have been conducted to develop alternative technologies to imaging-based technologies such that bone–implant integration can be accurately monitored. Most analyzed technologies are not yet validated in vivo (in animal models or in human patients), which would demonstrate their real potential when integrated into biological systems. Moreover, their effectiveness must be validated in comparison with imaging methods. Therefore, one cannot yet draw conclusions about their effectiveness. Further research is required to overcome the current limitations of the technologies stated herein. Moreover, technologies requiring extracorporeal components provide a limited monitoring capability, although their limitations can be overcome by miniaturizing and incorporating the components inside the implants. Indeed, an important capability that none of the technologies are able to provide (and just few referred to) is the possibility to monitor targeted regions, such that the overall time-dependent loosening states along the implant interface can be accurately identified. As the more critically unstable regions are detected, locally preventive treatments can be timely provided, thus decreasing the risk for revision surgeries. One must also highlight that capacitive technologies (already proposed for the strain methodology) hold potential for effective monitoring during the daily living of patients. As their co-surface architectures can be adapted to different topological structures and easily miniaturized, their design can be optimized to provide capacitive changes according to different bone–implant interface states. Their incorporation within endoprosthetic implants can provide a promising solution to optimize the monitoring capability of a wide range of bone–implant interfaces.

## Figures and Tables

**Table 1:** Comparative analysis of the monitoring methods to fulfill the effectiveness criteria. ^*a*,*b*^

Methodologies	Fixation	Methods	Requirements ^*c*^					
			(1)	(2)	(3)	(4)	(5)	(6)
Vibrometric	Cementless	Ext. Mechanical Excitation/Ext. Mechanical Signal	✓	✗	✗	✓	✗	✓
		Ext. Magnetic Induction/Ext. Mechanical Signal	✓	✓	✗	✓	✗	✓
		Int. Mechanical Excitation/Ext. Mechanical Signal	✓	✗	✗	✓	✗	✓
	Cemented	Ext. Mechanical Excitation/Ext. Mechanical Signal	✓	✗	✗	✓	✗	✓
		Ext. Mechanical Excitation/Int. Mechanical Signal	✓	✓	✗	✓	✗	✓
Acoustic	Cementless	Ext. Mechanical Excitation/Ext. Acoustic Signal	✓	✗	✗	✓	✗	✓
		Int. Mechanical Excitation/Ext. Acoustic Signal	✓	✗	✗	✓	✗	✓
		Ext. Magnetic Induction/Ext. Acoustic Signal	✓	✓	✗	✓	✗	✓
	Cemented	Ext. Mechanical Excitation/Ext. Acoustic Signal	✓	✗	✗	✓	✗	✓
		Int. Mechanical Excitation/Ext. Acoustic Signal	✓	✗	✗	✓	✗	✓
		Int. Mechanical Excitation/Int. Acoustic Signal	✓	✓	✗	✓	✗	✓
		Ext. Acoustic Emission/Ext. Acoustic Signal	✓	✗	✗	✓	✗	✗
Bioelectric Impedance	Cementless	Ext. Electrical Current/Ext. Electric Potential Difference	✓	✗	✗	✓	✗	✓
Magnetic Induction	Cementless	Ext. Magnetic Induction/Ext. Magnetic Induction	✓	✓	✗	✓	✗	✓
Strain	Cementless	Int. Mechanical Loads/Int. Bone Deformation	✗	✓	✓	✓	✗	✓
		Int. Mechanical Loads/Int. Fixation Plate Deformation	✓	✓	✓	✓	✗	✓

^*a*^ This table was built in such a way that if a method fulfills a criterion, at least one of their related technologies fulfill the criterion. ^*b*^ Terminology: Int.: Intracorporeal; Ext.: Extracorporeal. ^*c*^ Description: (1) Operate noninvasively regarding peri-implant tissues (2) Allow integration inside implants. (3) Allow stretchable and flexible integration inside implants. (4) Allow their design with different topological structures and for different geometries of the bone–implant interface. (5) Enable controllable and personalized monitoring of target regions on the tissues. (6) Allow follow-up of the bone–implant interface state throughout the daily life of patients.

## Appendix A. Technology Features Based on the Vibrometric Approach

**Table A1:** Technology features based on the vibrometric approach for cementless fixations. ^*a*^

	Refs.	Input	Output	Components and Location	Detection Aalgorithm	Monitoring Ability	In Vitro Validation	In Vivo Validation	Periodicity
**Method 1**	Georgiou and Cunningham [32]	Mechanical Vibration (<1 kHz)	Mechanical Vibration	Shaker: Knee; Accelerometer: Hip	Number of Harmonics and Amplitudes	Location: ND; States: Loose or Secure	—	23 Patients	Limited to Laboratory
	Alshuri et al. [33,34]	Mechanical Vibration (100–1500 Hz)	Mechanical Vibration	Shaker: Femoral Lateral Condyle; 2 Accelerometers: Iliac Crest and Greater Trochanter	Harmonic Ratios	Location: ND; States: Secure and 2 Loosening States	3 Composite Hemi-Pelvis; a Sawbones Femur	—	Limited to Laboratory
	Rieger et al. [35]	Mechanical Vibration (100–2000 Hz)	Mechanical Vibration	Shaker: Knee; 3 Accelerometers: Medial Condyle, Greater Trochanter, Ilium’s Crest	Frequency Shifts	Location: ND; States: Loose or Secure	Sawbone Femur and Hip	—	Limited to Laboratory
	Rieger et al. [36]	Mechanical Shock Waves	Mechanical Vibration	Piezoelectric Atuators: Lateral Knee Condyle, Rreater Trochanter, Ilium Crest; 3 Accelerometers: Same Locations	Frequency Shifts	Location: ND; States: Loose or Secure	3 Human Hip Specimens	—	Limited to Laboratory
	Lannocca et al. [37]; Varini et al. [38]	Mechanical Vibration (1.2–2 kHz)	Mechanical Vibration	Piezoelectric Actuator: Device; Accelerometer: Greater Trochanter	Frequency Shifts	Location: ND; States: Stable or Unstable	5 Femurs ^*b*^	—	Intra- Operatively
	Lannocca et al. [37]; Varini et al. [38]	Mechanical Vibration (1.2–2 kHz)	Mechanical Vibration	Piezoelectric Actuator: Device; LVDT: Greater Trochanter	Micro-Motions	Location: ND; States: Stable or Unstable	9 Femurs ^*b*^	—	Intra- Operatively
	Pastrav et al. [39]	Mechanical Vibration (<10 kHz)	Mechanical Vibration	Shaker: Prosthesis Neck; Mechanical Impedance Head: Prosthesis Neck	Frequency Shifts	Location: ND; States: Stable or Unstable	—	30 Patients	Intra- Operatively
	Jiang, Lee and Yuan [40]	Flexion- Extension Motion	Mechanical Vibration	Isokinetic Dynamometer: Leg (tibia); Accelerometer: Patella;	Spectral Power Ratios	Location: ND; States: Loose or Secure	—	14 Patients	Limited to Laboratory
**Method 2**	Ruther et al. [41,42]	Magnetic Induction (70 Hz)	Mechanical Vibration	Magnetic Oscilator: Inside the Implant (Porcine Ulna); Coil: 30 mm Apart of the Implant; Accelerometer: Porcine Foreleg	Frequency Shifts; Central Frequencies; Transient Periods	Location: ND; States: Secure and 2 Loosening States	7 Prcine Foreleg	—	Limited to Laboratory
**Method 3**	Glaser et al. [43]	Implat Motion	Mechanical Vibration	Two Accelerometers: Greater Trochanter and Iliac Spine	Output Signal High Frequency and Amplitude	Location: ND; States: Different Loosening States	—	5 Patients	Limited to Laboratory

^*a*^ Terminology: ND: not described; ^*b*^ Lannocca et al. [37] Provided Experimental Results Using Four Composite Femurs; Varini et al. [38] Presented Validations Results Using 5 Femurs (4 Cadaveric and 1 Composite).

**Table A2:** Technology features based on the vibrometric approach for cemented fixations. ^*a*^

	Refs.	Input	Output	Components and Location	Detection Algorithm	Monitoring Ability	In Vitro Validation	In Vivo Validation	Periodicity
**Method 1**	Li, Jones and Gregg [46]	Mechanical Vibration (100–1200 Hz)	Mechanical Vibration	Shaker: Distal Femur; Two Accelerometers: Distal and Proximal Femur	Frequency Shifts and Number of Harmonics	Location: ND; State: Secure or Loose	54 Models ^*b*^	—	Limited to Laboratory
	Rosenstein et al. [47]	Mechanical Vibration (100–1000 Hz)	Mechanical Vibration	Shaker: Lateral Condyle; Accelerometer: Greater Trochanter	Number of Harmonics	Location: ND; State: Secure or Loose	5 Femurs	11 Patients ^*c*^	Limited to Laboratory
	Rowlands, Duck and Cunningham [48]	Mechanical Vibration (100–1500 Hz)	Mechanical Vibration	Vibrator: Distal Femur; Accelerometer: Greater Trochanter	Harmonic Ratios	Location: ND; States: Secure or Loose	Sawbone Femur and Tufnol Tubing	—	Limited to Laboratory
	Leuridan et al. [49]	Mechanical Vibration	Mechanical Vibration	Impact Hammer: Tibial Plate; Accelerometers: Femur’s Surface and Tibial Plate	Frequency Response Function of the Output Signal	Location: ND; States: Secure and 3 Loosening Stages	5 Artificial Femurs	—	Limited to Laboratory
	Arami et al. [50]	Mechanical Vibration (30–3000 Hz)	Mechanical Vibration	Accelerometer and Vibrator: 10 cm Below the Patella; Two Accelerometers: Fixed to the Tibial Plate	Appearance of a New Peak in the Output Signal ^*d*^	Location: ND; State: Secure or Loose	14 Lower Limbs Specimens and 14 Cadavers	—	Limited to Laboratory
**Method 2**	Puers et al. [51]	Mechanical Vibration (100–200 Hz)	Mechanical Vibration	Shaker: Distal Femur; Accelerometer: Implant’s Femoral Head	Non-Similarity Between the Input and Output Signal	Location: ND; State: Secure or Loose	1 Cadaver	—	Limited to Laboratory
	Marschner et al. [52]	Mechanical Vibration (500–2500 Hz)	Mechanical Vibration	Shaker: Distal Femur; 2 Accelerometers: Distal End of Stem	Shifts in the Output Resonance Frequency (≈300 Hz)	Location: ND; State: Proximally Loose and Proximally Secure	1 Artificial Femur	—	Limited to Laboratory
	Sauer et al. [53]	Mechanical Vibration (500–2500 Hz)	Mechanical Vibration	Shaker: Central Femur; Accelerometer: Implant’s femoral Head	Shifts in the Output Resonance Frequency (20–100 Hz)	Location: ND; State: Three Loosening States	Artificial Femur	—	Limited to Laboratory

^*a*^ Terminology: ND: Not Described; ^*b*^ Twenty-One of the Secure Specimens, Seventeen of Loose and 16 of Early Loosening; ^*c*^ Seven of These were Admitted to a Revision Surgery and Four had done a Primary Total Hip Replacement Two Weeks Previously; ^*d*^ In the 750 to 900 Hz Range.

## Appendix B. Technology Features Based on the Acoustic Approach

**Table A3:** Technology features based on the acoustic approach for cementless fixations. ^*a*^

	Refs.	Input	Output	Components and Location	Detection Algorithm	Monitoring Ability	In Vitro Validation	In Vivo Validation	Periodicity
**Method 1**	Alshuri et al. [33,34]	Mechanical Vibration (100–1500 Hz)	Acoustic Waves	Shaker: Femoral Lateral Condyle; Ultrasound Probe: Iliac Crest	Harmonic Ratios	Location: ND; States: Secure and 2 Loosening States	3 Composite Hemi-Pelvis; a Sawbones Femur	—	Limited to Laboratory
	Unger et al. [55]	Mechanical Vibration	Acoustic Waves	Metallic Object: Implant Surface; Microphone: Lateral Femoral Condyle	Shifts in the Output Frequency	Location: ND; States: Different Loosening Stages	1 Cadaver	—	Limited to Laboratory
	Goosens et al. [56]	Mechanical Vibration	Acoustic Waves	Hammer; Microphone: 20 cm above the Experimental Setup	Shifts in the Output Frequency	Location: ND; States: Different Loosening Stages	Artificial Bone Block; Artificial Pelvis; and Cadaveric Pelvis	—	Limited to Laboratory
**Method 2**	Glaser et al. [43,57]	Implant’s Motion	Acoustic Waves	Acoustic Transducer: Hip’s Skin Surface	Output Signal High Frequency and Amplitude	Location: ND; States: Different Loosening Stages	—	29 Patients ^*b*^	Limited to Laboratory
**Method 3**	Ewald et al. [58,59]	Magnetic Induction	Acoustic Waves	Extracorporeal Coil; Microphone; Oscillators: Stem Walls	Shifts in the Output Frequency	Location: ND; States: Secure and 3 Loosening Stages	Bench Top Test Simulating Different Tissues Layers	—	Limited to Laboratory

^*a*^ Terminology: ND: Not described; ^*b*^ 24 patients in one study [57], and 5 in the other [43].

**Table A4:** Technology features based on the acoustic approach for cemented fixations. ^*a*^

	Refs.	Input	Output	Components and Location	Detection Algorithm	Monitoring Ability	In Vitro Validation	In Vivo Validation	Periodicity
**Method 1**	Rowlands, Duck and Cunningham [48]	Mechanical Vibration (100–1500 Hz)	Acoustic Waves	Vibrator: Distal Femur; Ultrasound Probe: Proximal Femur	Harmonic Ratios	Location: ND; States: Different Loosening Stages	Sawbone Femur and Tufnol Tubing	—	Limited to Laboratory
	Unger et al. [55]	Mechanical Vibration	Acoustic Waves	Hammer: Femoral Condyle; Microphone: Hip	Shifts in the Resonance Frequency	Location: ND; States: Loose or Secure	1 Cadaver	—	Limited to Laboratory
	Dahl et al. [60]	Mechanical Vibration	Acoustic Waves	Electromagnetic Actuator: Extracorporeal; Ultrasound Probe: Surface	Presence of Harmonics and Shifts in the Output Frequency	Location: ND; States: Loose or Secure	Cadaver Ankle	—	Limited to Laboratory
**Method 2**	Davies et al. [61]	Cement Degradation and Debonding	Acoustic Waves	Acoustic Emission Transducer: Femur’s Surface	Acoustic Emission Intensity and Output Signal Waveform	Location: ND; States: Several Loosening Stages	Artificial Femur (Fatigue Test—4 MPa at 2 Hz)	—	Limited to Laboratory
	Roques et al. [62]	Cement Cracks	Acoustic Waves	Two Accelerometers: Material’s Surface	Acoustic Emissions Energy and Signal Duration	Location: Could Detect; States: Different Interface States	Cement Blocks Specimens (Fatigue and 4 Point Bending Tests)	—	Limited to Laboratory
	Qi et al. [63]	Cement Cracks	Acoustic Waves	Eight Acoustic Sensors: Femur’s Surface	Acoustic Events Characteristics	Location: Could Detect; States: Different Interface States	Sawbones Femur (Fatigue Test)	—	Limited to Laboratory
	Gueiral and Nogueira [64]	Cement Cracks	Acoustic Waves	Three Acoustic Sensors: Femur’s Surface	Bursts of Acoustic Emissions	Location: Could Detect; States: Different Interface States	Sawbones Femur (Fatigue Test)	—	Limited to Laboratory
	Mavrogordato et al. [65]	Cement Cracks	Acoustic Waves	Acoustic Sensors: Externally Placed	Output Signal’s High Energy and Short Rise Time	Location: Could Detect; States: Different Interface States	Tufnol Tubing	—	Limited to Laboratory
**Method 3**	Mavrogordato et al. [65]	Cement Cracks	Acoustic Waves	Acoustic Sensors: Embedded in the Stem	Output Signal’s High Energy and Short Rise Time	Location: Could Detect; States: Different Interface States	Tufnol Tubing	—	Limited to Laboratory
**Method 4**	Davies, Tse and Harris [66]	Acoustic Emission	Acoustic Waves	Specimen Surface ^*b*^	Amplitude and Time of Arrival of the Reflected Wave	Location: ND; States: Bonded or Debonded	Cement Slab, Artificial and Cadaver Femurs	—	Limited to Laboratory

^*a*^ Terminology: ND: Not described; ^*b*^ Cement slab, artificial, and cadaver femur.

## Appendix C. Technology Features Based on the Bioelectric Impedance Approach

**Table A5:** Technology features based on the biolectric impedance approach for cementless fixations. ^*a*^

	Refs.	Input	Output	Components and Location	Detection Algorithm	Monitoring Ability	In Vitro Validation	In Vivo Validation	Periodicity
**Method 1**	Arpaia et al. [69,70]	Voltage (Sinusoidal: 10–100 mV)	Biolectrical Impedance	Location: Skin Surface, Near the Implant	Variation of Impedance	Location: ND; Secure or Loose	Cow Long Bone	3 Patients with Percutaneous Implants	Limited to Laboratory

^*a*^ Terminology: ND: not described.

## Appendix D. Technology Features Based on the Magnetic Induction Approach

**Table A6:** Technology features based on the magnetic induction approach for cementless fixations. ^*a*^

	Refs.	Input	Output	Components and Location	Detection Algorithm	Monitoring Ability	In Vitro Validation	In Vivo Validation	Periodicity
**Method 1**	Ewald et al. [58]	Magnetic Induction	Magnetic Induction	Coil: Extracorporeally; Piezo-Crystal: Inside the Implant	Difference in the Output Signal Amplitude	Location: ND; States: Secure and Different States of Loosening	Artificial Bone	—	Limited to Laboratory
	Ruther et al. [71]	Magnetic Induction	Magnetic Induction	Coils: Extracorporeally; Oscillators: Inside the Implant	Different Oscillator Velocity ^*b*^	Location: ND; Secure and Different States of Loose	Test Bench Apparatus	—	Limited to Laboratory

^*a*^ Terminology: ND: Not Described; ^*b*^ Translated into an induced voltage.

## Appendix E. Technology Features Based on the Strain Approach

**Table A7:** Technology features based on the strain approach for cementless fixations. ^*a*^

	Refs.	Input	Output	Components and Location	Detection Algorithm	Monitoring Ability	In Vitro Validation	In Vivo Validation	Periodicity
**Method 1**	Burton, Sun and Lynch [72]	Mechanical Load	Strain	Circuit: Wrapped Around the Bone Surface	Shifts in the Output Resonance Frequency	Location: ND; States: Several Deformation Stages (Axial and Radial)	Polyurethane Femur Model	—	Limited to Laboratory
**Method 2**	McGilvray et al. [73]	Mechanical Load	Strain	Fixation Plate: Along the Femur; Sensor: Center of the Fixation Plate	Shifts in the Output Resonance Frequency	Location: ND; States: Secure and Different Loosening Stages	Ovine Osteotomy	14 Sheep (Ovine)	Limited to Laboratory

^*a*^ Terminology: ND: Not described.
